# Integrated Analysis of mRNA and miRNA Expression Profiles in the Ovary of *Oryctolagus cuniculus* in Response to Gonadotrophic Stimulation

**DOI:** 10.3389/fendo.2019.00744

**Published:** 2019-10-29

**Authors:** Shenqiang Hu, Xiaohu Liang, Xufang Ren, Yu Shi, Hang Su, Yanhong Li, Kun Du, Jie Wang, Xianbo Jia, Shiyi Chen, Songjia Lai

**Affiliations:** Farm Animal Genetic Resources Exploration and Innovation Key Laboratory of Sichuan Province, Sichuan Agricultural University, Chengdu, China

**Keywords:** rabbit, gonadotrophin, follicle development and ovulation, microRNA, transcriptome sequencing

## Abstract

Molecular mechanisms responsible for gonadotrophic control of ovarian follicle development and ovulation have not been fully delineated. In this study, prepubertal female rabbits were subjected to a combined PMSG/hCG treatment for the induction of follicle maturation and ovulation. Ovaries of 6 does at different time points during gonadotrophic stimulation were collected for histomorphological examination and genome-wide analysis of miRNA and mRNA transcriptomes, and the plasma were separated for detecting melatonin (MT), prostaglandin E_2_ (PGE_2_), estradiol (E_2_), and progesterone (P_4_) levels. The results suggested that PMSG promoted the development of the reproductive tract by decreasing plasma levels of E_2_ and slightly increasing those of MT and PGE_2_ and that hCG induced ovulation and corpus luteum formation by significantly increasing MT, PGE_2_, and P_4_ levels. At the transcriptomic level, a total of 1,122 differentially expressed genes (DEGs) and 12 DE miRNAs were identified using three-group comparisons. Meanwhile, pairwise comparisons revealed that 279 and 103 genes as well as 36 and 20 miRNAs were up- and down-regulated during PMSG-stimulated follicle development while 11 and 5 genes as well as 33 and 16 miRNAs were up- and down-regulated during hCG-induced luteinization. KEGG enrichment analysis of the DEGs derived from both three-group- and two-group comparisons as well as the predicted target genes of DE miRNAs highlighted the crucial roles of pathways involving tissue remodeling, energy metabolism, and regulation of cellular functions in mediating gonadotrophin-induced follicle maturation. Specifically, 3 genes including the matrix metallopeptidase 13 (*MMP13*), protein phosphatase 1 regulatory subunit 3C (*PPP1R3C*), and solute carrier family 2 member 12 (*SLC2A12*), together with 2 miRNAs including the miR-205-1 and miR-34c, were predicted to be the promising downstream targets of both PMSG and hCG. Significantly, the miRNA-mRNA interaction pairs containing top 10 up- and down-regulated mRNAs/miRNAs upon PMSG/hCG stimulation were established, and so were those involved in the PI3K-Akt, ECM-receptor interaction, and focal adhesion pathways during PMSG-induced follicle maturation. Finally, qRT-PCR analysis confirmed the results from RNA-Seq and Small RNA-Seq. Our work may contribute to a better understanding of the regulatory mechanisms of gonadotrophins on ovarian follicle development and ovulation.

## Introduction

Paramount activities of the mammalian ovary are characterized by steroid hormone production as well as follicle maturation and ovulation, which are coordinately regulated by a number of factors emanating from extra-ovarian tissues and the ovary *per se* (1). With regard to the factors of extra-ovarian origin, adenohypophyseal hormones [e.g., follicle-stimulating hormone (FSH) and luteinization hormone (LH)] play pivotal roles in regulating ovarian activities (2). In support of this, the canonical two-cell/two-gonadotrophin mechanism controlling follicular steroidogenesis has been demonstrated in the mammalian ovary, where LH directs the androgen synthesis of theca cells while estrogens are aromatized from androgens in FSH-regulated granulosa cells. Furthermore, the preovulatory LH surge promotes the luteinization of granulosa cells, which then acquire the ability to secrete progesterone (3). Thus, it is of great value to unravel the mechanisms by which gonadotrophins orchestrate mammalian follicle development and ovulation.

The past century has seen a great advance in understanding such mechanisms. Although primordial follicle activation and preantral follicle development are independent of FSH but rely on factors secreted by the oocyte and granulosa cells, FSH is indispensable for the development of immature follicles into a preovulatory phenotype via activation of several downstream signaling cascades in granulosa cells, including the classical Gα_s_/adenylyl cyclase (AC)/cyclic AMP (cAMP)/protein kinase A (PKA), inositol triphosphate (IP3)/diacylglycerol (DAG)-dependent protein kinase C (PKC), and phosphoinositide 3-kinase (PI3K)/protein kinase B (Akt) pathways (4, 5). Cooperation of these pathways induces developmental stage-specific expression patterns of target genes related to granulosa cell proliferation (e.g., *CREB, CCND2*, and *CDKs*), differentiation (e.g., *EGFs, BMPs*, and *TGFs*), and steroidogenesis (e.g., *LHR, CYP11A1*, and *CYP19*) as well as formation and expansion of a fluid-filled antrum (e.g., *AQPs* and *HAS2*) (6–8). By contrast, LH triggers ovulation and luteinization of preovulatory follicles by downregulating cell proliferation-related genes (e.g., *FSHR, IGF1*, and *CCND2*) and upregulating genes related to cumulus cell expansion (e.g., *HAS2, PGS2*, and *COX2*), follicular rupture (e.g., *PR, ADAMTS1, CTSL*, and *MMPs*), and granulosa cell luteinization (e.g., *STAR, CYP11A1, LHR*, and *C/EBP*β), which are mainly mediated through the extracellular signal-regulated kinase 1/2 (ERK1/2) pathway in a cAMP/PKA-dependent manner (9, 10). These findings suggest that gonadotrophins modulate follicle development and ovulation by inducing both developmental stage- and cell type-specific expression of numerous genes related to different cellular functions depending on the complex interactions of multiple signaling cascades. Nevertheless, it remains a huge challenge to depict the global gonadotrophin-stimulated gene expression profile changes during follicle development and ovulation and to decipher the signaling networks.

In addition to protein-coding genes, microRNAs (miRNAs), a class of endogenous ~22 nt-long noncoding RNAs, also regulate follicle development and ovulation, since they are widely expressed in various ovarian cell types and regulate oogenesis and folliculogenesis by targeting a myriad of genes related to cellular proliferation, differentiation, luteinization, etc. (11–13). Rabbit (*Oryctolagus cuniculus*) not only serves as an economically important animal by providing superior meat, wool, and fur products but also an experimental animal model in biomedical researches. However, to date, information on global changes in the landscape of miRNA expression as well as on the miRNA-mRNA interaction networks during gonadotrophin-stimulated follicle development and ovulation remains scarce in rabbits. To achieve this, in the present study, the dynamic process of follicle maturation and ovulation was *in vivo* reproduced in sexually immature rabbits using validated hormonal treatments able to promote antral follicle development (PMSG injection) and ovulation (hCG treatment). Subsequently, gonadotrophin-stimulated changes in circulating hormones, ovarian morphology, and histology as well as mRNA and miRNA transcriptomes profiling were evaluated through the application of enzyme linked immunosorbent assay (ELISA), haematoxylin and eosin (H&E) staining, next-generation sequencing (NGS), and quantitative real-time PCR (qRT-PCR) techniques.

## Materials and Methods

### Ethics Statement

All experimental procedures involving the use of animals were conducted according to the “Guidelines for Experimental Animals” of the Ministry of Science and Technology (Beijing, China). This study was reviewed and approved by the Institutional Animal Care and Use Committee (IACUC) of Sichuan Agricultural University, under the permit No. DKY-B20141401.

### Animal Manipulation and Sample Collection

Sixty healthy and sexually immature females of Tianfu black rabbit (a Chinese indigenous rabbit breed with superior meat quality, Sichuan, China), aged 60 d and having similar body weights, were used in this study. The hormonal protocol used for induction of follicle maturation and ovulation was depicted in Supplementary Figure 1. Briefly, all does were kept at cages under a controlled light regimen of 14 L: 10 D, had free access to food and water, and were allowed to acclimate for 10 days before gonadotrophic administrations. At the age of 70 d, does were firstly injected intramuscularly with 100 IU of pregnant mare serum gonadotrophin (PMSG; Ningbo Second Hormone Factory, Zhejiang, China), and were then administered with 100 IU of human chorionic gonadotrophin (hCG; Ningbo Second Hormone Factory, Zhejiang, China) through marginal ear vein after 72 h of PMSG treatment. Six does were randomly selected and slaughtered at each time point, including just before PMSG injection (Control), 24 and 72 h after PMSG injection (P24 and P72), and 12 and 48 h after hCG administration (H12 and H48). Blood samples were collected with ethylenediaminetetraacetic acid (EDTA) vacutainers, and plasma was separated after centrifugation at 3000 × g for 15 min and then stored at −20°C until further analysis. After slaughter, ovaries were promptly removed and washed with 0.9% physiological saline, with the left ovary being used for histology and the right one for RNA extraction. Body weight and reproductive tract weight were recorded at all the time points.

### Histological Observation

All left-side ovaries were 4% formaldehyde-fixed for 72 h at room temperature, dehydrated through a graded ethanol series, transferred to xylene, and embedded in paraffin-wax. Paraffin sections of 5 μm thickness from each ovary were stained with H&E and photographed under a Nikon 90i microscope (Nikon, Japan). Ovarian follicles were generally classified into primordial, primary, secondary, tertiary, and mature follicles.

### Measurement of MT, PGE_2_, E_2_, and P_4_

Concentrations of plasma melatonin (MT), prostaglandin E_2_ (PGE_2_), estradiol (E_2_), and progesterone (P_4_) were determined according to the manufacturer's instructions using MT, PGE_2_, E_2_, and P_4_ ELISA kits (USCN Life Science Inc., Wuhan, Hubei, China). The actual sensitivities of MT, PGE_2_, E_2_, and P_4_ are typically <4.63 pg/ml, <8.43 pg/ml, <4.45 pg/ml, and <0.55 ng/ml, respectively. The average recovery rates of MT, PGE_2_, E_2_, and P_4_ are around 95, 98, 92, and 94%, respectively. The mean intra- and inter-assay coefficients of variation for each of MT, PGE_2_, E_2_, and P_4_ were <10 and <12%, respectively. Serial dilutions of plasma samples showed good linearity (differed by 20% or less) in the calculated concentrations of either MT, or PGE_2_, or E_2_, or P_4_. Each of MT, PGE_2_, E_2_, and P_4_ had no significant cross-reactivity or interference with respective analogs.

### Total RNA Isolation

Total RNA was extracted from the right-side ovaries using Trizol reagent (Invitrogen, Carlsbad, CA, USA) and treated with DNase I (Invitrogen, Carlsbad, CA, USA) following the manufacturers' protocol. The RNA concentration, purity, and integrity number (RIN) were determined using a NanoDrop spectrophotometer (Thermo Fisher Scientific, Waltham, MA, USA) and an Agilent 2100 Bioanalyzer (Agilent Technologies, CA, USA), with the RNA concentration >434 ng/μl in a final volume of 35 μl and a range of RIN between 9.0 and 10.0. Thereafter, two RNA samples from each of the Control, P72, and H48 groups were randomly pooled in equal quantities to generate three RNA preparations per group, each of which was then divided into two aliquots processed for construction of either RNA-Seq or Small RNA-Seq library.

### RNA Sequencing and Analysis

The mRNA was isolated from total RNA using poly-T oligo attached magnetic beads (Invitrogen, Carlsbad, CA, USA), followed by purification and interruption into short fragments with the fragmentation buffer. Subsequently, 9 cDNA libraries for RNA sequencing were prepared with an Illumina TruSeq RNA Sample Preparation Kit (Illumina, San Diego, CA, USA) according to the manufacture's instruction. Then, all libraries were sequenced on an Illumina Hiseq x-ten platform that generated >6G 150-bp paired-end (PE) raw reads per sample by Beijing Genome Institute (BGI, Shenzhen, China). After sequencing, the raw reads were evaluated with FastQC software and filtered by removing adaptor sequences as well as contamination and low quality reads with >5% unknown bases and a quality score <20%. Then, the clean reads were mapped to the rabbit reference genome OryCun2.0 (https://www.ncbi.nlm.nih.gov/assembly/GCF_000003625.3) using HISAT2 (v2.0.0), and the Bowtie2 (v2.2.9) software was further used to map the reads to the reference genes for better control of sequencing quality.

The abundance of each gene was expressed as the number of clean reads mapped to its sequence that was normalized to fragments per kilobase of exon per million fragments mapped (FPKM) using RSEM (v1.2.30). Differentially expressed genes (DEGs) in two-group comparisons were screened using DESeq2 (v1.16.1), and the cutoff for significant differential expression was |log2 fold change (FC)| ≥ 1 with a false discovery rate (FDR) <0.05. DEGs in three-group comparisons were screened using the TCC package (v1.20.0) in R (v3.4.4) software, and a Bayesian method called BaySeq was applied to estimate significant differential expression with a FDR of 0.1 and an iteration of 1 (14). Time-series expression pattern analysis was performed by the Short Time-series Expression Miner (STEM) (v1.3.11) software using the STEM Clustering Method, with “*Maximum Number of Model Profiles*” and “*Maximum Unit Change in Model Profiles between Time Points*” set at 50 and 2, respectively (15). Hierarchical clustering and principle component analysis (PCA) were conducted in R (v3.4.4) software. Gene Ontology (GO) and Kyoto Encyclopedia of Genes and Genomes (KEGG) enrichment analyses of the selected DEGs were performed using the Database for Annotation, Visualization, and Integrated Discovery (DAVID) v6.8 (http://david.abcc.ncifcrf.gov/), with an EASE score at 0.05.

### Small RNA Sequencing and Analysis

Nine small RNA cDNA libraries were prepared with the TruSeq Small RNA Sample Preparation Kit (Illumina, San Diego, CA, USA) following the manufacture's instruction. All library preparations were checked for quality using an Agilent 2100 Bioanalyzer (Agilent Technologies, CA, USA) and then were sequenced on an Illumina Hiseq 4000 platform that generated >10 M 50-bp single-end (SE) raw reads per sample by Beijing Genome Institute (BGI, Shenzhen, China). After removal of adaptors, junk, and low quality sequences, the clean reads with the length of 18–30 nucleotides were mapped to the rabbit reference genome using Bowtie2 (v2.2.9) combined with the Rfam database to identify different categories of small RNAs.

Identification of known miRNAs and prediction of novel miRNAs were performed with the miRBase v22 database (http://www.mirbase.org/) and miRDeep2 software, respectively. The expression level of each miRNA was expressed as the tags per million reads (TPM) using DESeq2, and significantly differentially expressed miRNAs (DE miRNAs) in two-group comparisons were identified under the criteria of |log2FC| ≥ 1 and FDR <0.05. DE miRNAs in three-group comparisons were screened using the TCC package (v1.20.0) in R (v3.4.4) software, and BaySeq was applied to estimate significant differential expression with a FDR of 0.1 and an iteration of 1 (14). The target genes of DE miRNAs were predicted using miRanda and RNAhybrid with respective default parameters, and their overlapping gene list was used for GO and KEGG analyses. Finally, the miRNA-mRNA pairs involving PMSG/hCG-induced follicle development and ovulation as well as specific signaling pathways were identified according to their anti-correlated expression profiles, and their interaction networks were depicted using Cytoscape (v3.7.1) software.

### Validation Experiments

For qRT-PCR analysis and validation of our sequencing data, total RNA were isolated from 30 ovarian tissue samples containing the Control, P24, P72, H12, and H48 groups (*n* = 6/group). 100 ng of total RNA were reversed transcribed using the PrimeScript™ RT reagent Kit with gDNA Eraser (Takara Biotechnology Co., Ltd., Dalian, China) according to the manufacture's instruction. The PCR reactions were performed on the CFX96™ Real-Time PCR Detection System (Bio-Rad, USA) using the SYBR Premix Ex Taq™ II (Takara Biotechnology Co., Ltd., Dalian, China). Reactions were conducted with the following conditions: pre-denaturation at 95°C for 5 min, followed by 40 cycles of denaturation at 95°C for 15 s and annealing/extension at corresponding temperature of each primer set for 30 s. The no-template controls and negative controls without reverse transcriptase were also included in all qPCR runs. Target specificity for each primer set was validated by melting curve analyses, and the identity of all amplicons was verified by sequencing. All samples were amplified in triplicate and relative expression levels of target genes were normalized to the reference gene *GAPDH* using the comparative Cq method (ΔΔCq) (16).

First-strand cDNA of miRNA was synthesized from total RNA using the Mir-X™ miRNA First-Strand Synthesis Kit (Takara Biotechnology Co., Ltd., Dalian, China) according to the manufacture's instruction, and quantification of miRNAs of interest was also performed on the CFX96™ Real-Time PCR Detection System (Bio-Rad, USA) using the Mir-X™ miRNA qRT-PCR SYBR^®^ Kit (Takara Biotechnology Co., Ltd., Dalian, China). The PCR reactions were conducted under a similar condition to target gene expression detection. Relative expression levels of target miRNAs were normalized to the reference gene *U6 snRNA* using the ΔΔCq method (16). The qRT-PCR primers of selected genes and miRNAs are listed in Supplementary Table 1.

### Statistical Analyses

All data were expressed as mean ± SEM. Statistical comparisons between groups were analyzed by analysis of variance (ANOVA) followed by Tukey's test using SAS 9.4 (SAS Institute, Cary, USA). A *p* < 0.05 was considered statistically significant.

## Results

### Histomorphological Responses to Gonadotrophic Stimulation in Prepubertal Female Rabbits

**Figure 1:**
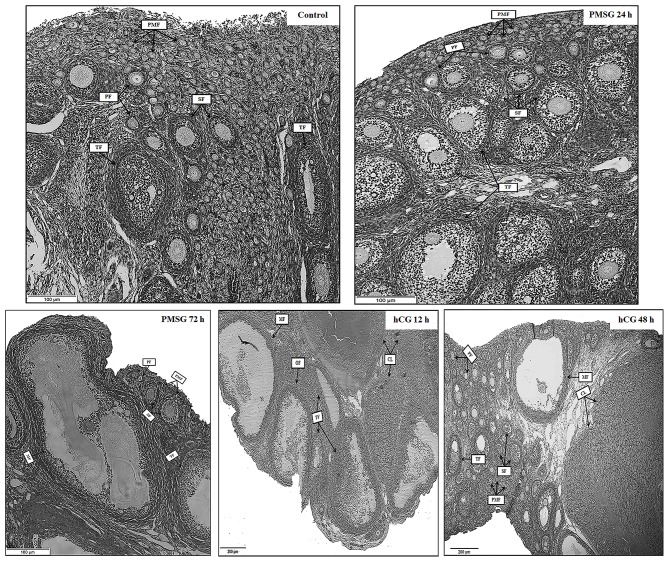
Ovarian histology of prepubertal female rabbits upon gonadotrophic stimulation. Ovaries were collected from the Control, P24, P72, H12, and H48 groups, respectively. PMF, primordial follicle; PF, primary follicle; SF, secondary follicle; TF, tertiary follicle; MF, mature follicle; OF, ovulating follicle; CL, corpus luteum; Scale bar: 100 or 200 μm.

Significant morphological and histological changes were seen in the reproductive tracts of does after hormone administrations, as shown in Table 1 and Figure 1. In detail, gonadotrophic stimulation had no significant effect on body weight, fallopian tube weight, and the proportion of fallopian tube weight to body weight of prepubertal does (*P* > 0.05), but it is notable that both fallopian tube weight and its proportion to body weight increased gradually from Control to P72 and reached the maxima in H12. An around 3- to 5-fold increase in both ovary weight and its proportion to body weight occurred after 72 h of PMSG treatment, and their highest levels were observed at 12 h following hCG treatment (*P* < 0.05). For uterus weight and its proportion to body weight, both increased continuously from Control to H48, reaching the maxima in H48 (*P* < 0.05). H&E staining of ovarian sections revealed that both the composition and number of follicles of different size categories differed before and after hormone injection. Compared to the Control group whose ovaries were only composed of primordial, primary, secondary, and tertiary follicles, mature follicles appeared at 72 h after PMSG injection until 48 h following hCG treatment, ovulating follicles were seen in the H12 group, and corpora lutea were present in both the H12 and H48 groups. Additionally, injection of PMSG increased the number of tertiary follicles in P24 and that of mature follicles in P72, while hCG treatment induced follicular rupture in H12 and increased the number of corpora lutea in H48.

**Table 1:** Body and reproductive organ weights of prepubertal does in response to gonadotrophic stimulation.

**Parameter**	**Control (*n* = 6)**	**PMSG 24 h (*n* = 6)**	**PMSG 72 h (*n* = 6)**	**hCG 12 h (*n* = 6)**	**hCG 48 h (*n* = 6)**
BW (kg)	2.1783 ± 0.0626^a^	2.0250 ± 0.0642^a^	2.0750 ± 0.0733^a^	2.0583 ± 0.0999^a^	1.9417 ± 0.0539^a^
OW (g)	0.1641 ± 0.0493^b^	0.1596 ± 0.0183^b^	0.6317 ± 0.0980^ab^	0.8488 ± 0.2562^a^	0.5091 ± 0.1009^ab^
OW/BW (g/kg)	0.0778 ± 0.0244^b^	0.0785 ± 0.0078^b^	0.3037 ± 0.0451^ab^	0.4275 ± 0.1402^a^	0.2577 ± 0.0433^ab^
FW (g)	0.2771 ± 0.0381^a^	0.3412 ± 0.0247^a^	0.3564 ± 0.0136^a^	0.3713 ± 0.0335^a^	0.2878 ± 0.0273^a^
FW/BW (g/kg)	0.1278 ± 0.0183^a^	0.1696 ± 0.0146^a^	0.1721 ± 0.0058^a^	0.1819 ± 0.0177^a^	0.1487 ± 0.0148^a^
UW (g)	1.1747 ± 0.1811^b^	1.2648 ± 0.1073^b^	2.6355 ± 0.5927^ab^	2.9015 ± 0.5289^ab^	3.4247 ± 0.5682^a^
UW/BW (g/kg)	0.5427 ± 0.0851^b^	0.6271 ± 0.0545^b^	1.2578 ± 0.2747^ab^	1.3882 ± 0.2179^ab^	1.7741 ± 0.3035^a^

*BW, body weight; OW, bilateral ovary weight; FW, bilateral fallopian tube weight; UW, bilateral uterus weight. Values were expressed as Mean ± SEM. Different lowercase letters in each row indicated statistically significant differences among groups at P < 0.05*.

### Plasma Hormonal Responses to Gonadotrophic Stimulation in Prepubertal Female Rabbits

**Figure 2:**
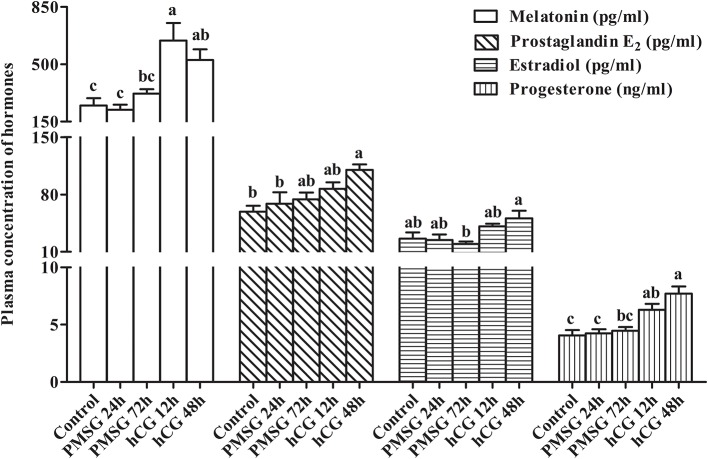
Changes in plasma concentration of reproduction-related hormones in prepubertal female rabbits upon gonadotrophic stimulation. Values were expressed as the mean ± SEM of six rabbits per group. Different lowercase letters indicated significant differences among groups at *P* < 0.05.

Changes in circulating levels of MT, PGE_2_, E_2_, and P_4_ were determined before and after gonadotrophic stimulation. As shown in Figure 2, plasma concentrations of MT increased significantly (*P* < 0.05) from Control (248.07 ± 44.83 pg/ml) to H12 (644.89 ± 107.44 pg/ml), but then decreased to 526.90 ± 64.48 pg/ml in H48. A continuous increase from Control to H48 was seen in plasma levels of PGE_2_, reaching the highest level (110.11 ± 6.61 pg/ml) in H48 (*P* < 0.05). However, levels of E_2_ decreased gradually (*P* > 0.05) from Control (26.17 ± 7.59 pg/ml) to P72 (19.55 ± 2.97 pg/ml), but then increased dramatically to 41.14 ± 3.08 pg/ml in H12 and peaked in H48 (50.87 ± 9.11 pg/ml; *P* < 0.05). Similar to PGE_2_, levels of P_4_ increased continuously from Control to H48 and reached the maxima (7.70 ± 0.63 pg/ml) in H48; and compared to the groups before P72, hCG administration significantly (*P* < 0.05) increased its levels in both H12 and H48.

### Mapping and Annotation of RNA Sequencing Data

In order to reveal the mRNA transcriptomic response to gonadotrophic stimulation, nine cDNA libraries representing rabbit ovaries from the control group (just before PMSG: C-M1, C-M2, C-M3) and two experimental groups (72 h after PMSG: P-M1, P-M2, P-M3; 48 h after hCG: H-M1, H-M2, H-M3) were constructed with respective mRNA pool and sequenced. As shown in Supplementary Table 2, the Q20 ratio, Q30 ratio, and GC content varied from 96.84 to 97.09, 91.90 to 92.47, and 51.89 to 54.69%, respectively, and 75.24–78.30% clean reads from each library were mapped to the reference rabbit genome. Analysis of distribution of clean reads on either chromosomes or reference genes showed that our RNA-Seq data had sufficient sequencing depth and coverage. Finally, a total of 20,719 expressed genes were identified among three groups, comprising 71.20% of all rabbit genes (29,098), with an average number of 18,597, 18,424, and 18,515 for the C, P, and H group, respectively.

### Dynamics of Rabbit Ovarian mRNA Transcriptome in Response to Gonadotrophic Stimulation

**Figure 3:**
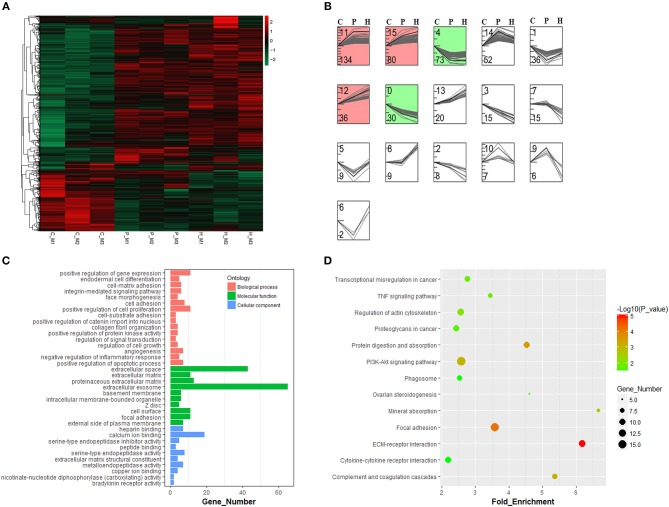
Hierarchical clustering and time-series modules of differentially expressed genes (DEGs) among nine libraries. **(A)** Heat map of the expression profile of DEGs screened by the TCC package in R focusing on multi-group data comparisons. **(B)** Time-series cluster analysis for gene expression profiles of DEGs. The STEM profile No. and the number of transcripts within each profile were shown at the top and down left corner, respectively. The red and green panels contained genes showing significantly increased and decreased expression levels through time, respectively. **(C)** Gene ontology and KEGG pathway **(D)** annotations of genes in both green and red panels.

As shown in Supplementary Figure 2, hierarchical clustering and PCA analyses showed that nine cDNA libraries were sorted into three distinct clusters (i.e., C, P, and H) corresponding to respective treatment and that relatively higher similarity in the expression profiles of all expressed genes was found among three cDNA libraries from the same group, which was indicative of good reproducibility of high-throughput sequencing. After standardization of raw data using the Trimmed Mean of M-values (TMM) method, 1122 DEGs were obtained among the C, P, and H groups using TCC in R. Hierarchical clustering analysis suggested that gonadotrophic treatment had a significant effect on the expression profiling of these DEGs and that two distinct profiles were present before and after PMSG stimulation (Figure 3A). The STEM results showed that these 1122 DEGs were categorized into 16 profiles according to their temporal expression patterns during follicular maturation and ovulation. Among the top seven profiles ranked by decreasing number of genes, profiles 11, 15, and 12 represented a total of 250 genes, whose expression were significantly unregulated through time; while profiles 0 and 4 contained 103 genes, presenting a significant time-dependent decrease in the mRNA levels (Figure 3B).

Three hundred fifty-three genes up- or down-regulated through time were annotated to three main GO categories, including biological process, molecular function, and cellular component. For biological process, most genes were enriched in regulation of gene expression, cell proliferation, cell adhesion, apoptotic process, and inflammatory response as well as angiogenesis and morphogenesis. For molecular function, most enriched terms included extracellular exosome and extracellular space. For cellular component, most enriched terms included calcium ion binding and endopeptidase activity (Figure 3C). KEGG enrichment analysis revealed that most of these 353 genes were mainly enriched in the PI3K-Akt signaling, focal adhesion, regulation of actin cytoskeleton, ECM-receptor interaction, cytokine-cytokine receptor interaction, phagosome, TNF signaling, and ovarian steroidogenesis pathways (Figure 3D).

**Figure 4:**
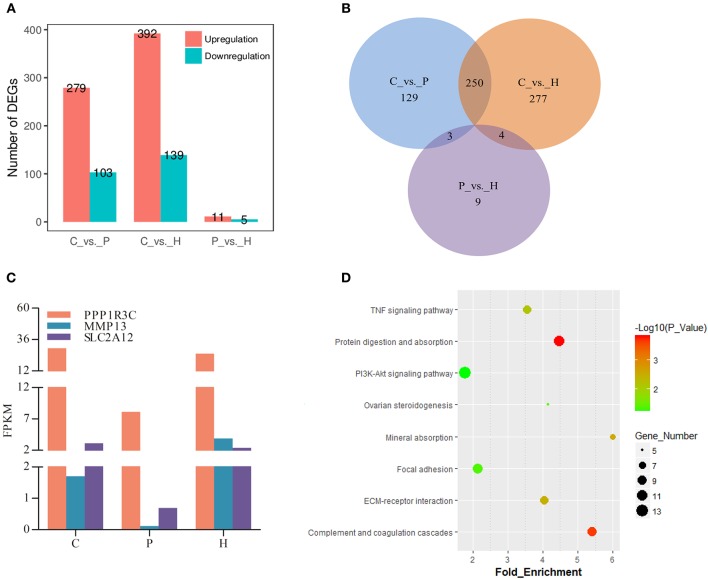
Identification and analysis of DEGs in rabbit ovaries upon gonadotrophic stimulation. **(A)** The number of up/down-regulated genes in each pairwise comparison group. **(B)** Venn diagram of DEGs between three pairwise comparisons. **(C)** Dynamic expression profile of three identified overlapping DEGs between CP and PH comparisons in rabbit ovaries before and after gonadotrophic treatment. **(D)** KEGG enrichment analysis of DEGs merged from three comparison groups.

In addition to three-group comparison, we also performed pairwise comparisons among the C, P, and H groups using DESeq2 in R. The results showed that a total of 531 DEGs were identified between C and H, 279 up- and 103 down-regulated genes were identified in C vs. P, and 11 and 5 genes were up- and down-regulated in P vs. H, respectively (Figure 4A). In particular, the top 10 up- and down-regulated DEGs upon PMSG stimulation were shown in Supplementary Table 3, with the serpin family B member 2 (*SERPINB2*) and proteolipid protein 1 (*PLP1*) being the most up- and down-regulated gene, respectively. Supplementary Table 4 listed all significantly regulated DEGs following hCG administration, showing that hCG increased expression of liver carboxylesterase (*LOC100009551*), matrix metallopeptidase 13 (*MMP13*), prostaglandin F_2α_ receptor (*PTGFR*), secreted frizzled related protein 4 (*SFRP4*), hydroxyprostaglandin dehydrogenase 15-(NAD) (*HPGD*), neuromedin B *(NMB*), ependymin related 1 (*EPDR1*), protein phosphatase 1 regulatory subunit 3C (*PPP1R3C*), the adiponectin C1Q and collagen domain containing (*ADIPOQ*), docking protein 5 (*DOK5*), and solute carrier family 2 member 12 (*SLC2A12*) but decreased that of aldo-keto reductase family 1 member C1 (*AKR1C5*), ectonucleotide pyrophosphatase/phosphodiesterase 6 (*ENPP6*), ribonuclease 8 (*LOC100352281*), endoplasmic reticulum-Golgi intermediate compartment protein 2 (*LOC100354670*), and the BPI fold-containing family B member 4 gene (*LOC100357983*). A Venn diagram showed that no common DEGs were present in all three pairwise comparisons, whereas, FPKM of 3 genes (i.e., *PPP1R3C, MMP13*, and *SLC2A12*) were significantly altered by both PMSG and hCG treatments (Figures 4B,C). KEGG analysis suggested that the representative pathways enriched by these DEGs included the PI3K-Akt signaling, protein digestion and absorption, complement and coagulation cascade, ECM-receptor interaction, focal adhesion, TNF signaling, mineral absorption, and ovarian steroidogenesis (Figure 4D). Particularly, there were 8 DEGs enriched in ovarian steroidogenesis, including *STAR, 3*β*HSD, CYP19A1*, adenylate cyclase 8 (*ADCY8*), cytochrome P450 1B1 (*CYP1B1*), bone morphogenetic protein 15 (*BMP15*), *BMP6*, and prostaglandin-endoperoxide synthase 2 (*PTGS2*).

### Mapping and Annotation of Small RNA Sequencing Data

To further identify global changes in the miRNA transcriptome before and after gonadotrophic stimulation, nine small RNA libraries were constructed using total RNAs extracted from the same rabbit ovaries for mRNA-Seq and sequenced. As shown in Supplementary Table 5, the Q20 ratio of each library was more than 99.63%, and the GC content ranged from 44.43 to 45.27%. The length distribution of clean reads was similar among all libraries, presenting that most reads had 21–23 nt in length, with the 22 nt RNAs being the most abundant. It was observed that 89.54–91.11% clean reads from each library were mapped to the reference rabbit genome. An average number of 591, 592, and 595 known miRNAs together with 325, 326, and 334 novel miRNAs were identified in the C, P, and H group, respectively.

### Dynamics of Rabbit Ovarian miRNA Transcriptome in Response to Gonadotrophic Stimulation

**Figure 5:**
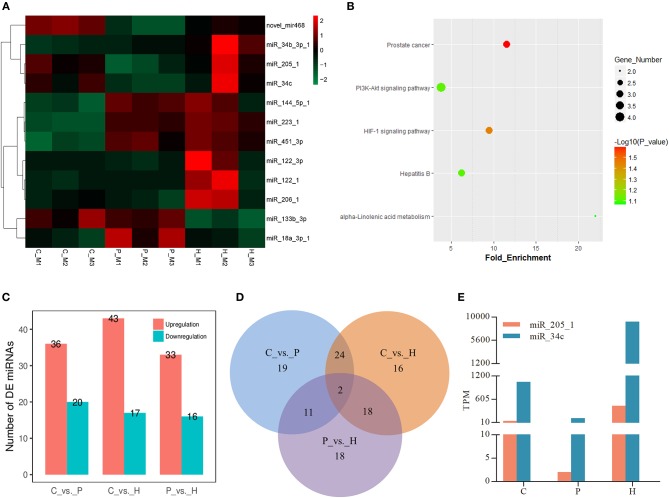
Expression profiles of differentially expressed (DE) miRNAs in rabbit ovaries upon gonadotrophic stimulation. **(A)** Heat map of the expression profile of DE miRNAs screened by the TCC package in R focusing on multi-group data comparisons. **(B)** KEGG enrichment analysis of the predicted target genes of DE miRNAs through time. **(C)** The number of up/down-regulated miRNAs in each pairwise comparison group. **(D)** Venn diagram of DE miRNAs between three pairwise comparisons. **(E)** Dynamic expression profile of 2 identified overlapping DE miRNAs among three pairwise comparisons in rabbit ovaries before and after gonadotrophic treatment.

Similar to the mRNA transcriptome analysis, 12 DE miRNAs were identified among the C, P, and H groups using TCC in R. Hierarchical clustering analysis verified that expression of these DE miRNAs significantly changed before and after gonadotrophic treatment (Figure 5A). KEGG enrichment analysis of the predicted target genes of these DE miRNAs showed that their targets were mainly enriched in the PI3K-Akt signaling, HIF-1 signaling, prostate cancer, hepatitis B, and alpha-linolenic acid metabolism (Figure 5B).

In addition to three-group comparison, we also performed pairwise comparisons among C, P, and H using DESeq2 in R. The results showed that a total of 60 DE miRNAs were identified between C and H, 36 up- and 20 down-regulated miRNAs were found in C vs. P, and 33 and 16 miRNAs were up- and down-regulated in P vs. H, respectively (Figure 5C). In particular, the top 10 up- and down-regulated DE known miRNAs following either PMSG or hCG administration were listed in Supplementary Tables 6, 7. Of note, miR-205-1 and miR-34c were not only in the list of 12 DE miRNAs screened by three-group comparison, but they were also identified to be significantly differentially expressed in any of three pairwise comparisons (Figures 5A,D). Furthermore, TPM of these two miRNAs decreased following 72 h of PMSG treatment, but later on, increased by hCG (Figure 5E).

### Integrative Analysis of DE mRNAs and DE miRNAs

Based on the predicted target genes of DE miRNAs screened by small RNA-Seq and the DE mRNAs resulted from RNA-Seq, significantly altered expression of both miRNAs and their predicted target mRNAs were identified upon either PMSG or hCG administration, and so were DE mRNAs and their regulatory miRNAs. Supplementary Figure 3 showed the top 10 overlapping pairs between the predicted target genes of top 10 down/up-regulated miRNAs and DE mRNAs in response to PMSG and hCG administration. As the two most downregulated known miRNAs after PMSG injection, the top 10 matched target genes of miR-34b included galectin 9 (*LGALS9*), fibroblast growth factor-binding protein 1 (*LOC100354095*), tenascin C (*TNC*), leukemia inhibitory factor (*LIF*), suppressor of cytokine signaling 3 (*SOCS3*), kinase suppressor of ras 1(*KSR1*), ADAM metallopeptidase with thrombospondin type 1 motif 15 (*ADAMTS15*), dual specificity phosphatase 4 (*DUSP4*), proteoglycan 4 (*PRG4*), and mesenteric estrogen dependent adipogenesis (*MEDAG*), while those of miR-205-5p included parathyroid hormone-like hormone (*PTHLH*), collagen type VIII alpha 1 (*COL8A1*), dipeptidase 1 (*DPEP1*), *LGALS9, TNC*, solute carrier family 12 member 3 (*SLC12A3*), *LOC108177736*, heme oxygenase 1 (*HMOX1*), *LIF*, and pyrimidinergic receptor P2Y6 (*P2RY6*). By contrast, as for the top 2 upregulated miRNAs by PMSG, one- (coagulation factor XIII A chain, *LOC103352261*) and four-matched target genes (sperm associated antigen 17, *SPAG17*; double homeobox protein A-like, *LOC100355249*; cytohesin 1 interacting protein, *CYTIP*; *LOC103349621*) were identified for miR-451-3p and miR-7b, respectively. Additionally, the top 10 matched target genes of miR-205-1 upon PMSG stimulation included *PTHLH, COL8A1, DPEP1, LGALS9*, interferon regulatory factor 7 (*IRF7*), 2′-5′-oligoadenylate synthase-like protein (*LOC100356242*), *TNC*, urokinase plasminogen activator receptor (*PLAUR*), *SLC12A3*, and matrilin 4 (*MATN4*), while those of miR-34c included serpin family E member 1 (*SERPINE1*), *LGALS9, LOC100354095, TNC, SLC12A3*, C-C motif chemokine receptor 2 (*CCR2*), *LIF, P2RY6, SOCS3*, and *KSR1*. By comparison, only one matched target gene for either miR-205-1 or miR-34c was found upon hCG administration, and moreover, both target was *LOC100357983*.

**Figure 6:**
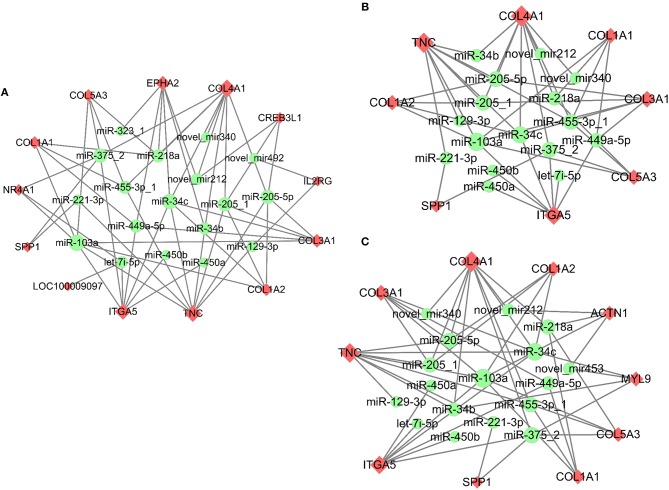
Co-expression network analysis of the miRNA-mRNA interaction pairs involved in the PI3K-Akt **(A)**, ECM-receptor interaction **(B)**, and focal adhesion **(C)** pathways in response to PMSG stimulation. Upregulated mRNAs were shaded in pink, while downregulated miRNAs were shaded in green. The shape size of each mRNA indicated the number of miRNAs regulating its expression, while that of each miRNA indicated the number of its targeted mRNAs.

Meanwhile, the predicted miRNAs modulating expression of the top 10 up/down-regulated DE mRNAs that matched DE miRNAs in response to PMSG and hCG stimulation were also summarized in Supplementary Figure 4. Similarly, the up/down-regulated target mRNAs were well matched with the down/up-regulated miRNAs. For instance, hCG administration reduced expression of both miR-199a-5p-1 and miR-22-5p but enhanced that of their common target gene namely *PTGFR*. Significantly, following 72 h of PMSG treatment, the miRNA-mRNA interaction pairs enriched in the PI3K-Akt signaling, ECM receptor interaction, and focal adhesion pathways were further identified according to their anti-correlated expression profiles, respectively (Figure 6). Among these pairs, it is noticeable that expression of the DEGs increased upon PMSG stimulation but levels of their regulatory miRNAs decreased. Of particular note was the simultaneous involvement of secreted phosphoprotein 1 (*SPP1*), integrin subunit alpha 5 (*ITGA5*), *TNC*, collagen type I alpha 1 (*COL1A1*), collagen type I alpha 2 (*COL1A2*), collagen type III alpha 1 (*COL3A1*), collagen type IV alpha 1 (*COL4A1*), and collagen type V alpha 3 (*COL5A3*) in these three pathways, which were regulated by a cluster of miRNAs, respectively (Figure 6).

### Correlation Between qRT-PCR and Illumina Sequencing

**Figure 7:**
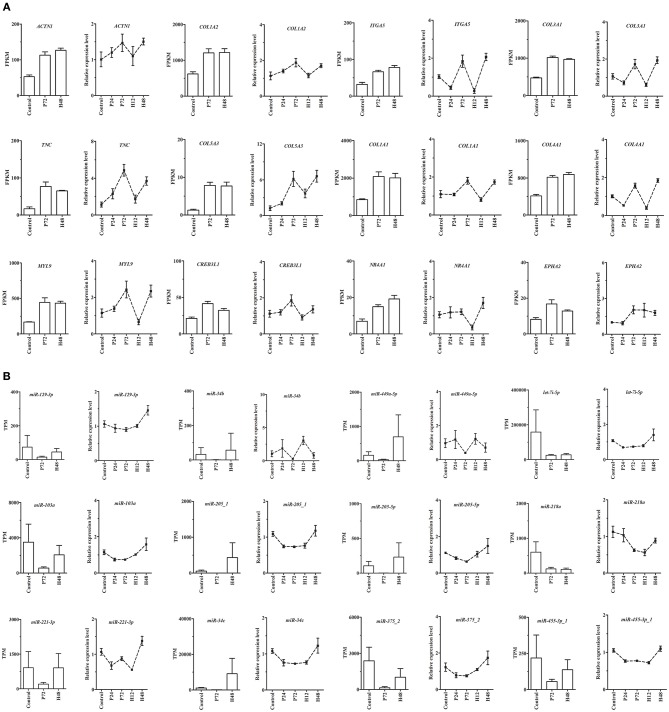
qRT-PCR validation of expression of the main mRNAs **(A)** and miRNAs **(B)** involving the PI3K-Akt, ECM-receptor interaction, and focal adhesion pathways in rabbit ovaries before and after gonadotrophic stimulation. RNA-seq was performed only in the Control, P72, and H48 groups, and the values were expressed as the mean ± SEM of 3 pooled ovaries per group. In contrast, qRT-PCR was performed in all five groups, and the values were expressed as the mean ± SEM of 6 individual ovaries per group. The RNA-seq and qRT-PCR results were depicted as the bar and line charts, respectively.

From DE mRNAs and DE miRNAs screened by RNA-Seq and small RNA-Seq, 9 genes (*MMP13, TIMP1, SLC2A12, PPP1R3C, RASD1, SPP1, ADIPOQ, ACSL6*, and *STAR*) and 6 miRNAs (miR-22-5p, miR-542-5p-1, miR-7b, miR-129a-3p, miR-30c-1-3p-1, and miR-34a-5p) were randomly selected for qRT-PCR validation (Supplementary Figure 5). Moreover, the main miRNA-mRNA pairs involved in the PI3K-Akt, ECM-receptor interaction, and focal adhesion pathways were also validated by qRT-PCR (Figure 7). The quantitative results showed that despite differences in the magnitude of fold-changes, expression of almost all these selected mRNAs and miRNAs determined by qRT-PCR displayed changes in the same direction with that observed by the sequencing data, indicating the true reliability of our RNA-Seq and small RNA-Seq methods. Additionally, expression of these selected mRNAs and miRNAs at other sampling points (i.e., P24 and H12) were also detected by qRT-PCR, providing a better understanding of their roles during follicle maturation and CL formation.

## Discussion

Ovarian follicle maturation and ovulation are achieved by gonadotrophic stimulation (2). However, the mechanisms of gonadotrophins regulating these two events have not been fully deciphered. Herein, the effects of combined PMSG/hCG administrations on plasma hormone levels as well as ovarian histomorphology and mRNA and miRNA transcriptomes were systematically evaluated in sexually immature does. Similar to the observations that in primiparous and multiparous does a combination of PMSG and hCG used for either estrus synchronization or superovulation markedly stimulated follicular growth and ovulation (17, 18), we demonstrated that PMSG was effective in stimulating the reproductive tract development, as manifested by an increase in its weight and the number of mature follicles, and moreover, ovulation of PMSG-treated does was triggered with hCG. At the plasma level, E_2_ reached the minima at 72 h after PMSG treatment, while hCG significantly increased MT and P_4_ levels of PMSG-treated does. Furthermore, PMSG/hCG combinations significantly increased PGE_2_ levels. MT is mainly secreted by the pineal gland and regulates follicle development and ovulation by affecting both adenohypophyseal hormone release and ovarian steroid production (19). PGE_2_, E_2_, and P_4_ are primarily synthesized by ovarian follicles (20, 21), and among them, PGE_2_ is recognized as a key mediator in gonadotrophin-stimulated ovulation and fertilization (22) while E_2_ and P_4_ are indispensable for follicle and CL development (2). Thus, it was postulated that follicle maturation is mainly associated with altering levels of E_2_ and PGE_2_ while rising MT, PGE_2_, and P_4_ levels induce ovulation and CL formation.

To reveal the underlying molecular mechanisms, we determined genome-wide profiling of ovarian mRNA and miRNA expression. Results from three-group- and pairwise-comparisons identified 1122 and 672 DEGs throughout follicle development and ovulation, respectively, and 353 of 1,122 DEGs were further screened by STEM analysis. Since GO classification and KEGG enrichment analysis are widely used in the annotation of gene functions (23, 24), these 353 DEGs were further annotated to be mainly enriched into the BP terms associated with regulation of gene expression, cellular functions, inflammatory process, and face morphogenesis. Meanwhile, both these 353 DEGs and those 672 DEGs were enriched into the PI3K-Akt signaling, focal adhesion, protein digestion and absorption, complement and coagulation cascades, ECM-receptor interaction, TNF signaling, mineral absorption, and ovarian steroidogenesis pathways, most of which are known to be associated with regulation of cellular functions and tissue remodeling (25–28). For instance, those 8 DEGs enriched in the “ovarian steroidogenesis” pathway have been demonstrated to regulate ovarian steroid production in rabbits or other mammals (29–32). Similarly, three DEGs identified during hCG-induced luteinization, including *HPGD, PTGFR*, and *AKR1C5*, were reported to be important for CL formation by regulating steroid hormone and prostaglandin metabolism (3, 22, 29, 30). Hence, we proposed that differential expression of these DEGs could be responsible for gonadotrophin-stimulated plasma hormonal fluctuations, thereby promoting rabbit ovarian follicle development and ovulation. As for those three genes regulated by both PMSG and hCG treatments, *MMP13* is crucial for ECM remodeling and follicular-luteal transition (33), overexpression of *PPP1R3C* increased hepatic gluconeogenesis and glycogen storage by targeting protein phosphatase 1 (34), and *SLC2A12* was reported to modulate glucose utilization in chicken skeletal muscles (35), supporting the roles of ECM remodeling and glucose metabolism during follicle maturation and ovulation. However, the physiological significance of *PPP1R3C* and *SLC2A12* in the mammalian ovary awaits further investigations. These data suggest that gonadotrophin-stimulated follicle development and ovulation are finely tuned by a wide array of genes related to steroidogenesis, morphogenesis, and functional differentiation of ovarian cells.

Numerous miRNAs have been characterized to regulate follicle maturation and ovulation in a range of domestic animals (12, 36, 37). However, to the best of our knowledge, this study represents the first to depict global miRNAome changes in rabbit ovaries responding to gonadotrophic stimuli. The predicted target genes of the 12 DE miRNAs identified by three-group comparison were mainly enriched into the PI3K-Akt signaling, prostate cancer, HIF-1 signaling, hepatitis B, and alpha-linolenic acid metabolism pathways, which were evidenced to activate the transcription of genes related to angiogenesis, inflammation, energy metabolism, and cellular functions (25, 38, 39), indicating that these miRNAs play important roles in mediating the actions of gonadotrophins in the rabbit ovary. Among them, both miR-205-1 and miR-34c levels were reduced by PMSG but were enhanced by hCG. Since the miR-205 and miR-34 families were recognized as key regulators of cell survival, proliferation, differentiation, apoptosis, and cell cycle arrest in ovarian cancer cells (40, 41) and more than 10 predicted target genes of both miR-205-1 and miR-34c matched our mRNA-seq data upon PMSG stimulation but only one matched target gene for either one upon hCG stimulation, we speculated that both miR-205-1 and miR-34c may function differently between PMSG-stimulated follicle development and hCG-induced ovulation by targeting different genes. Besides, as the two most downregulated miRNAs by PMSG, miR-34b and miR-205-5p were reported to regulate cell apoptosis and cell-cycle arrest in many types of cancers, and among their common predicted target genes, *LGALS9, TNC*, and *LIF* are known as regulators of cell apoptosis, proliferation, and adhesion (42–44), suggesting that reduced levels of both miRNAs may promote PMSG-stimulated follicle maturation by regulating ovarian cellular proliferation and apoptosis. By contrast, less is known about the physiological functions of the two most unregulated miRNAs by PMSG as well as the two most up- and down-regulated miRNAs by hCG in the ovary.

The key miRNA-mRNA pairs involved in the PI3K-Akt, ECM-receptor interaction, and focal adhesion pathways were identified, because these pathways have essential roles in regulating PMSG-stimulated follicle maturation (26–28). Furthermore, expression of eight DEGs commonly enriched in these pathways and their predicted regulatory miRNAs were validated by qRT-PCR, showing reduced miRNAs levels in parallel with rising mRNAs levels upon PMSG stimulation. Among them, collagens and tenascins are known as components of ECM that promote and counteract follicular cell adhesion, respectively (45). SPP1 is an adhesion molecule that plays important roles in regulating developmental processes, cell-cell interactions, inflammatory responses, and carcinogenesis (46), and moreover, it promoted the progress of human ovarian cancer through the Integrin β1/FAK/AKT signaling pathway (47) and its expression was regulated by estrogen in both the porcine uterus (48) and the chicken oviduct (49). Integrins are cell surface receptors that mediates interactions between cells and surrounding ECM (45), and they were able to modulate cell cycle progression and cell proliferation via chk1 and Rb/E2F pathways and were also regulated by E_2_ in breast cancer cells (50, 51). Hence, we postulated that these eight DEGs are important for PMSG-stimulated follicle maturation by not only constituting follicular cell microenvironment but also regulating cellular functions through multiple signaling pathways, and their upregulated mRNA levels may be associated with a reduction in plasma E_2_ concentration. Nevertheless, the real roles of their regulatory miRNAs during mammalian follicle development remain largely unknown and need to be further studied. In addition, regarding that only 16 DEGs were identified during hCG-induced ovulation, the miRNAs predicted to regulate all these DEGs that matched DE miRNAs were fully identified. Among them, since *PTGFR* mediates PGF_2α_-induced luteolysis and parturition (22) and *MMP13* regulates follicular-luteal transition (33), it was inferred that miR-199a-5p-1 and miR-22-5p could target *PTGFR* while miR-542-5p, miR-107-1, and miR-30c-1-3p could target *MMP13* to regulate ovulation and CL formation.

We also recognized the limitations of using the rabbit as our experimental model because rabbits have distinct reproductive characteristics from humans. Specifically, compared to humans, rabbits are induced ovulators, do not have a pronounced reproductive cycle, and normally ovulate 8–12 oocytes (52). Nevertheless, rabbits share a similar process of gonadotrophin-stimulated follicle development and ovulation with humans and other domestic animals, which, together with the high consistency between the qRT-PCR results and transcriptome sequencing, highlight the importance and accuracy of our data in fully dissecting the regulatory mechanisms of gonadotrophins in the mammalian ovary. These identified DE mRNAs and miRNAs as well as their interaction networks also provide us a new avenue to reveal the molecular mechanisms controlling rabbit ovarian activities, which will be beneficial for improving reproductive efficiency in the rabbit industry.

## Data Availability Statement

The transcriptome sequencing data are available in the Sequence Read Archive (https://www.ncbi.nlm.nih.gov/sra) at NCBI, with the BioProject ID: PRJNA552172 and SRA Accession Number: SRR9639659-9639676.

## Ethics Statement

The animal study was reviewed and approved by Institutional Animal Care and Use Committee (IACUC), Sichuan Agricultural University.

## Author Contributions

SH, XL, and SL conceptualized and designed this study. SH, XL, and XR performed the experiments, analyzed the data, and drafted the manuscript. YS, HS, YL, KD, JW, XJ, and SC participated in animal manipulation, data collection, and analysis. SH reviewed this manuscript. SL supervised this study.

### Conflict of Interest

The authors declare that the research was conducted in the absence of any commercial or financial relationships that could be construed as a potential conflict of interest.

## Supplementary Material

Supplementary Figure 1 A schematic representation of hormonal regimen used for induction of follicle maturation and ovulation in prepubertal female rabbits. 60 does at 60 days of age were first injected with 100 IU of PMSG after 10 days of acclimation to stimulate antral follicle development, and 72 h after PMSG treatment were administered with 100 IU of hCG to induce ovulation and luteinization. Six does per group were randomly sacrificed just before (Control) and then 24 h (P24) and 72 h (P72) after PMSG treatment, as well as 12 h (H12) and 48 h (H48) after administration of hCG.

Supplementary Figure 2 Hierarchical clustering and principle component analysis (PCA) of the mRNA transcriptome among nine libraries. **(A)** Heat map of the expression profile of all identified mRNAs. Each row represented the relative levels of individual mRNA, while each column represented the expression level of a single sample. The top tree was built based on Pearson correlation coefficient. **(B)** PCA clustered the samples of similar gene expression profiles according to gonadotrophic stimulation.

Supplementary Figure 3 Co-expression network analysis of the top 10 down/up-regulated miRNAs and corresponding top 10 predicted target mRNAs that match DE mRNAs in response to PMSG **(A)** and hCG **(B)** stimulation. Upregulated mRNAs and miRNAs were shaded in pink, while downregulated mRNAs and miRNAs were shaded in green. The shape size of each mRNA indicated the number of miRNAs regulating its expression, while that of each miRNA indicated the number of its targeted mRNAs.

Supplementary Figure 4 Co-expression network analysis of the top 10 down/up-regulated mRNAs and corresponding top 10 predicted regulatory miRNAs that match DE miRNAs in response to PMSG **(A)** and hCG **(B)** stimulation. Upregulated mRNAs and miRNAs were shaded in pink, while downregulated mRNAs and miRNAs were shaded in green. The shape size of each mRNA indicated the number of miRNAs regulating its expression, while that of each miRNA indicated the number of its targeted mRNAs.

Supplementary Figure 5 qRT-PCR validation of expression of nine mRNAs **(A)** and six miRNAs **(B)** in rabbit ovaries before and after gonadotrophic stimulation. RNA-seq was performed only in the Control, P72, and H48 groups, and the values were expressed as the mean±SEM of three pooled ovaries per group. In contrast, qRT-PCR was performed in all five groups, and the values were expressed as the mean ± SEM of six individual ovaries per group. The RNA-seq and qRT-PCR results were depicted as the bar and line charts, respectively.

Supplementary Table 1 Primer pairs for quantitative real-time PCR.

Supplementary Table 2 Quality analysis and mapping of RNA-Seq clean reads to the rabbit reference genome.

Supplementary Table 3 Top 10 up/down-regulated DEGs following PMSG stimulation.

Supplementary Table 4 All up/down-regulated DEGs following hCG stimulation.

Supplementary Table 5 Categories of small RNA-Seq clean tags mapped to Rfam.

Supplementary Table 6 Top 10 up/down-regulated known miRNAs following PMSG stimulation.

Supplementary Table 7 Top 10 up/down-regulated known miRNAs following hCG stimulation.
