# Supplementary figures and images for: Integrated Analysis of mRNA and miRNA Expression Profiles in the Ovary of Oryctolagus cuniculus in Response to Gonadotrophic Stimulation
Source: Front Endocrinol (Lausanne). 2019 Oct 29;10:744. doi: 10.3389/fendo.2019.00744 (PMC6828822; doi:10.3389/fendo.2019.00744)

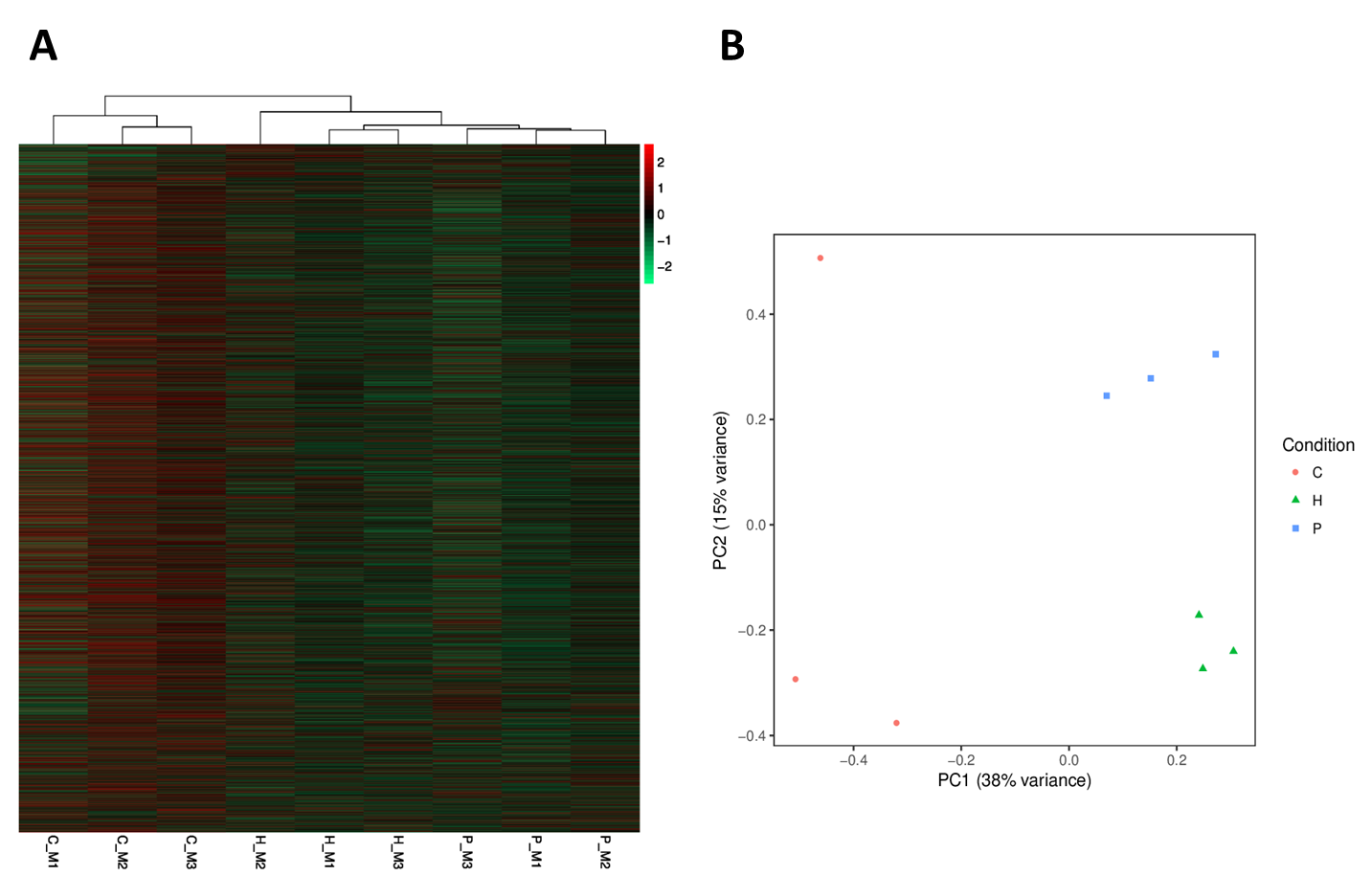

Supplement: Supplementary Figure 2 — Hierarchical clustering and principle component analysis (PCA) of the mRNA transcriptome among nine libraries. (A) Heat map of the expression profile of all identified mRNAs. Each row represented the relative levels of individual mRNA, while each column represented the expression level of a single sample. The top tree was built based on Pearson correlation coefficient. (B) PCA clustered the samples of similar gene expression profiles according to gonadotrophic stimulation. [file Image_2.TIF]

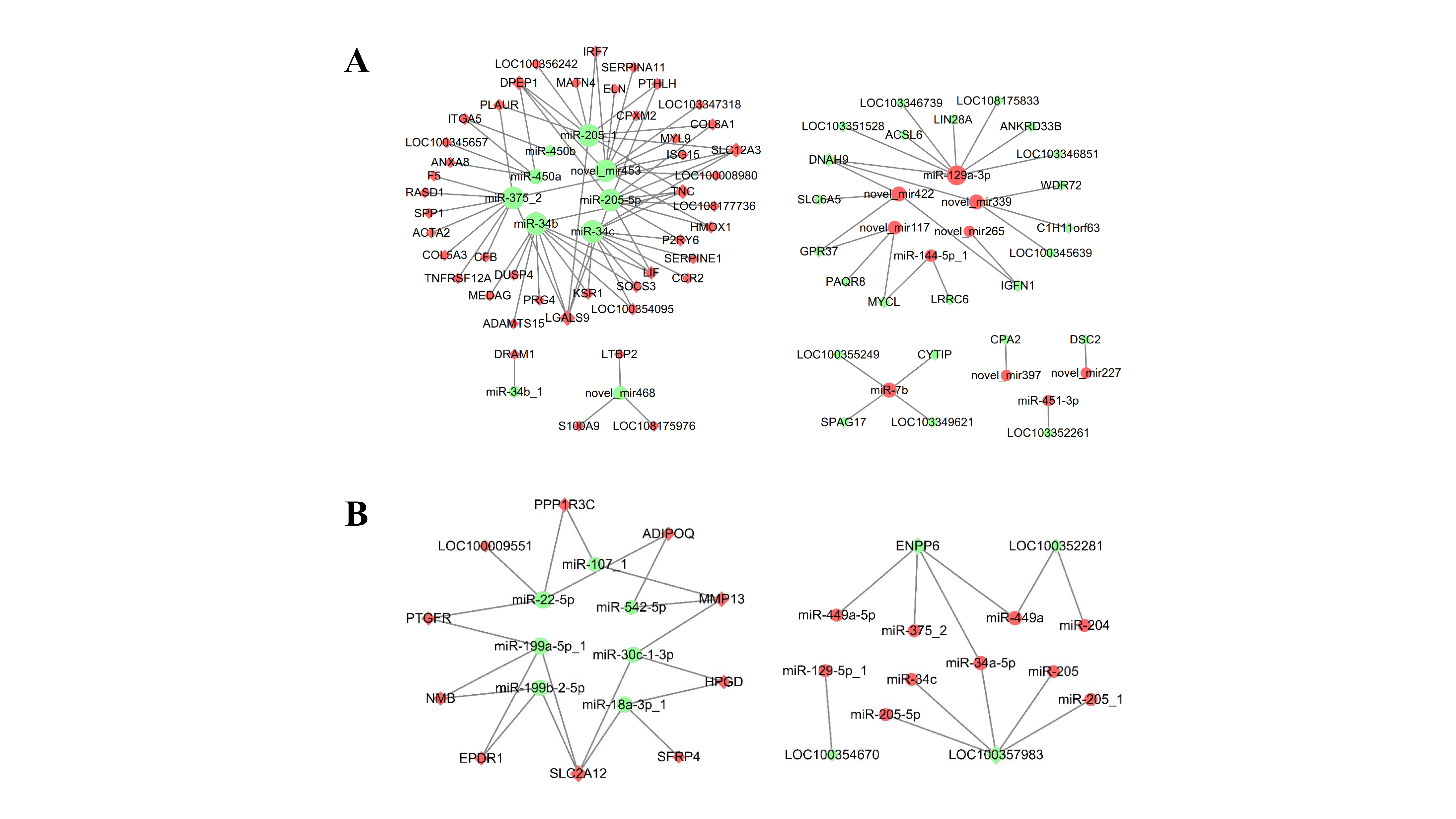

Supplement: Supplementary Figure 3 — Co-expression network analysis of the top 10 down/up-regulated miRNAs and corresponding top 10 predicted target mRNAs that match DE mRNAs in response to PMSG (A) and hCG (B) stimulation. Upregulated mRNAs and miRNAs were shaded in pink, while downregulated mRNAs and miRNAs were shaded in green. The shape size of each mRNA indicated the number of miRNAs regulating its expression, while that of each miRNA indicated the number of its targeted mRNAs. [file Image_3.TIF]

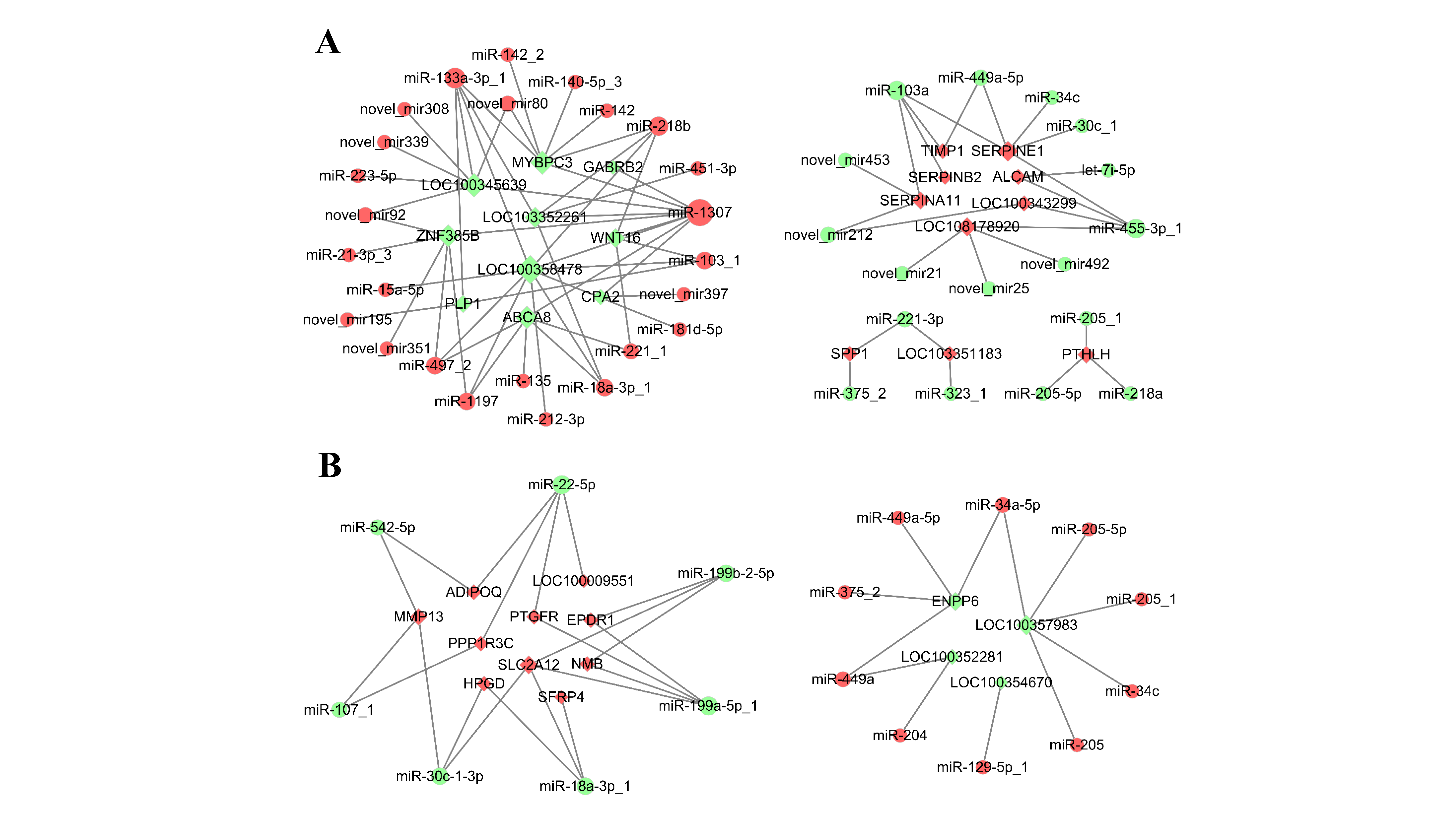

Supplement: Supplementary Figure 4 — Co-expression network analysis of the top 10 down/up-regulated mRNAs and corresponding top 10 predicted regulatory miRNAs that match DE miRNAs in response to PMSG (A) and hCG (B) stimulation. Upregulated mRNAs and miRNAs were shaded in pink, while downregulated mRNAs and miRNAs were shaded in green. The shape size of each mRNA indicated the number of miRNAs regulating its expression, while that of each miRNA indicated the number of its targeted mRNAs. [file Image_4.TIF]

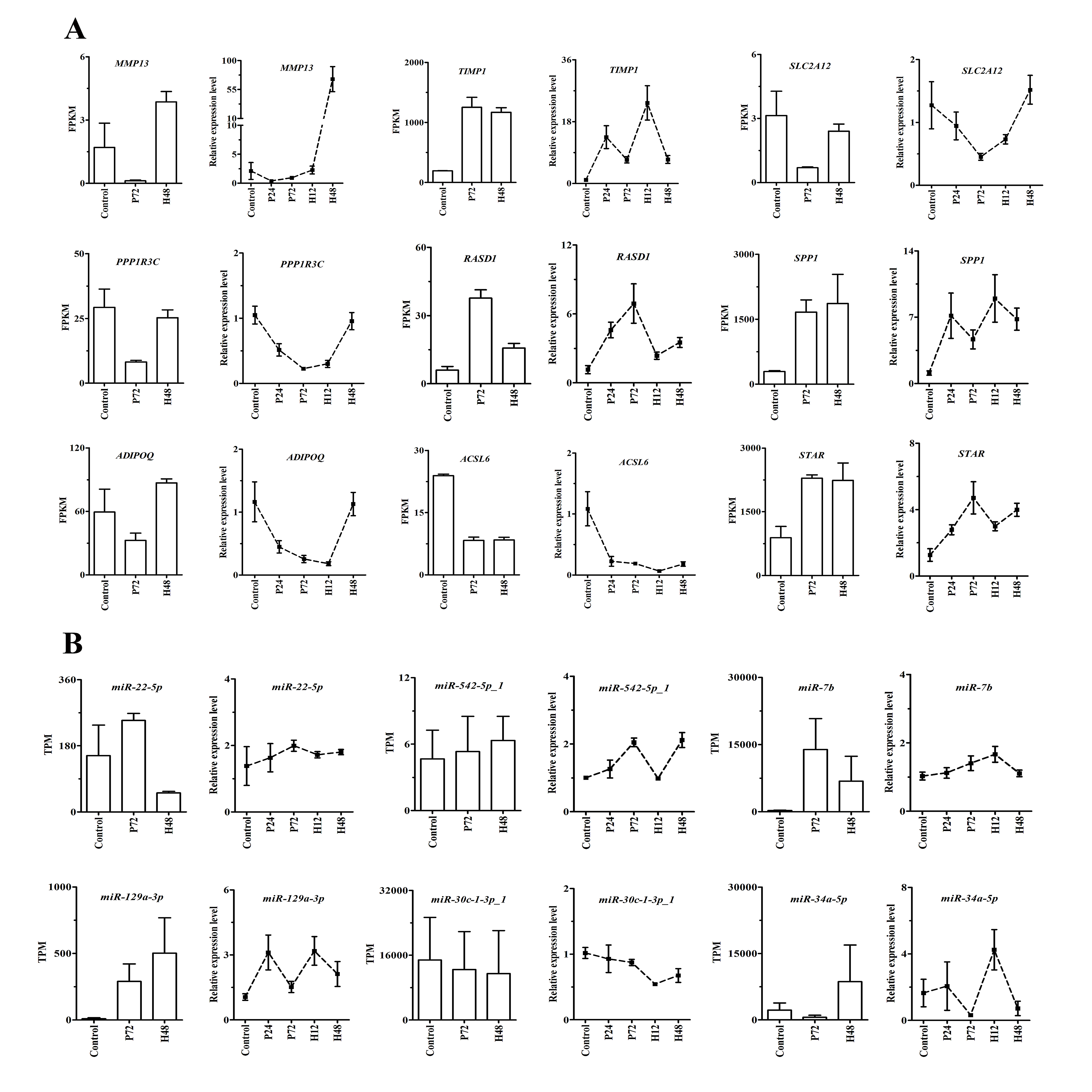

Supplement: Supplementary Figure 5 — qRT-PCR validation of expression of nine mRNAs (A) and six miRNAs (B) in rabbit ovaries before and after gonadotrophic stimulation. RNA-seq was performed only in the Control, P72, and H48 groups, and the values were expressed as the mean±SEM of three pooled ovaries per group. In contrast, qRT-PCR was performed in all five groups, and the values were expressed as the mean ± SEM of six individual ovaries per group. The RNA-seq and qRT-PCR results were depicted as the bar and line charts, respectively. [file Image_5.TIF]
