# Supplementary material for: Integrated Analysis of mRNA and miRNA Expression Profiles in the Ovary of Oryctolagus cuniculus in Response to Gonadotrophic Stimulation
Source: Front Endocrinol (Lausanne). 2019 Oct 29;10:744. doi: 10.3389/fendo.2019.00744 (PMC6828822; doi:10.3389/fendo.2019.00744)
Supplement: Supplementary Table 1 — Primer pairs for quantitative real-time PCR. [file Table_1.DOCX]

**Suppl. Table 1. Primer pairs for quantitative real-time PCR**

| **Primer name** | **Sequence (5’to 3’)** | | **Tm (℃)** | **Size (bp)** |
| --- | --- | --- | --- | --- |
| ***mRNAs*** | | | | |
| *COL1A2* | F | GATGGCTACCCAACTTGCCT | 59.0 | 207 |
|  | R | GTGCAGCCATCGACAAGAAC |  |  |
| *ITGA5* | F | CGGCTTCTCGGTGGAGTTTT | 55.7 | 198 |
|  | R | GACAGGGACGATTCCAGGAC |  |  |
| *COL3A1* | F | TGGAGTGGCTGGACCAAAAG | 61.4 | 123 |
|  | R | AGACCCCGAGCTCCTGTAAT |  |  |
| *TNC* | F | GGGGCAGTGTGTTCATCTCA | 61.4 | 185 |
|  | R | AATCAGCCCATGAAGGTGGG |  |  |
| *COL5A3* | F | GATGGTGGGGGTGAATCAGG | 63.3 | 149 |
|  | R | GGGCTGTGCTGGAATCTCTT |  |  |
| *COL1A1* | F | CACTCTGACTGGAAGAGCGG | 63.3 | 213 |
|  | R | GAACTGGAAGCCATCGGTCA |  |  |
| *COL4A1* | F | ACCAGGCACAGTCAAACGAT | 61.4 | 173 |
|  | R | TTGCAGAACAGGAAGGGCAT |  |  |
| *ACTN1* | F | TCCCGAGGAGTTCAAAGC | 58.0 | 179 |
|  | R | CCGCCGTATCGGTGTCT |  |  |
| *MYL9* | F | ACGTCCAATGTCTTCGCCAT | 63.3 | 154 |
|  | R | GGTATTCGTCCGTGGGGTTT |  |  |
| *EPHA2* | F | AAGGGGGACTCCAACAGCTA | 63.3 | 153 |
|  | R | CGTCTGGAACTCATGCACCT |  |  |
| *CREB3L1* | F | CTGAGTTCTCATCCGGCTCC | 59.0 | 110 |
|  | R | CGCCCCTTCATCGTAGAACA |  |  |
| *NR4A1* | F | CTGCACTCCTTGGTTGTGGA | 63.3 | 147 |
|  | R | GTCCCCCACGTGTTCCTTTA |  |  |
| *STAR* | F | TTCCGACTGGAGGTGGTGCTG | 63.3 | 147 |
|  | R | GTGCGTGATGACCGTGTCCTTC |  |  |
| *TIMP1* | F | ACACTCATTGCTTGTGGACAGACC | 61.0 | 150 |
|  | R | CTTCCAACAGGAGCAGCCTTCAG |  |  |
| *SLC2A12* | F | GGTCAAGGTTGTGAGCACCATCC | 57.0 | 80 |
|  | R | AGGAGCCGACGCACAGGAAG |  |  |
| *ADIPOQ* | F | AGGTGCCTATGTCTACCGCTCAG | 63.3 | 125 |
|  | R | CCGTGGTGCTGTCATAGTGGTTC |  |  |
| *PPP1R3C* | F | GCTCACTGCCATCCACGTCTTC | 55.7 | 94 |
|  | R | GCCAGAGGAGATGTCGTTCAAGTC |  |  |
| *ACSL6* | F | GGCGTGTCGGCTTCTTCCAG | 63.3 | 99 |
|  | R | GGTTCAGCAGTCGTGGCACTAC |  |  |
| *RASD1* | F | GGAGACGTGTTCATCCTGGTGTTC | 59.0 | 109 |
|  | R | TCTTGTTCTTGAGGCACGACTTGG |  |  |
| *SPP1* | F | GACTCCAATGAATCCGACGATGCC | 57.0 | 196 |
|  | R | GTCTGTAAGCCACACTGTCACCAC |  |  |
| *MMP13* | F | TCTACACCTACACCGGCAAGAGTC | 63.3 | 163 |
|  | R | CGGAGACTGGTAATGGCATCAAGG |  |  |
| *GAPDH* | F | CTTCGGCATTGTGGAGGG | 61.0 | 130 |
|  | R | GGAGGCAGGGATGATGTTCT |  |  |
| ***miRNAs*** | | | | |
| *miR-129-3p* | F | AAGCCCTTACCCCAAAAAGT |  |  |
| *miR-34b* | F | AGGCAGTGTAATTAGCTGATTG |  |  |
| *miR-449a-5p* | F | TGGCAGTGTATTGTTAGCTGG |  |  |
| *let-7i-5p* | F | TGAGGTAGTAGTTTGTGCTGT |  |  |
| *miR-103a* | F | AGCAGCATTGTACAGGGCTATGAAG |  |  |
| *miR-205_1* | F | TCCTTCATTCCACCGGAGTCTG |  |  |
| *miR-205-5p* | F | TCCTTCATTCCACCGGAGTCT |  |  |
| *miR-218a* | F | TTGTGCTTGATCTAACCATGTGGTT |  |  |
| *miR-221-3p* | F | AGCTACATTGTCTGCTGGGTTTC |  |  |
| *miR-34c* | F | AGGCAGTGTAGTTAGCTGATTG |  |  |
| *miR-375_2* | F | TTTTGTTCGTTCGGCTCGCGTGA |  |  |
| *miR-455-3p_1* | F | ATGCAGTCCATGGGCATATACA |  |  |
| *miR-22-5p* | F | AGTTCTTCAGTGGCAAGCTTTA |  |  |
| *miR-542-5p-1* | F | TCGGGGATCATCATGTCACGA |  |  |
| *miR-7b* | F | TGGAAGACTAGTGATTTTGTTGTTT |  |  |
| *miR-129a-3p* | F | AAGCCCTTACCCCAAAAAGTAT |  |  |
| *miR-30c-1-3p-1* | F | TGGGAGAGGGTTGTTTACTCC |  |  |
| *miR-34a-5p* | F | AGGCAGTGTAGTTAGCTGATTGC |  |  |
| *U6 snRNA* | F | GGAACGATACAGAGAAGATTAGC |  |  |
|  | R | TGGAACGCTTCACGAATTTGCG |  |  |

F, forward primer; R, reverse primer
