# Supplementary material for: Integrated Analysis of mRNA and miRNA Expression Profiles in the Ovary of Oryctolagus cuniculus in Response to Gonadotrophic Stimulation
Source: Front Endocrinol (Lausanne). 2019 Oct 29;10:744. doi: 10.3389/fendo.2019.00744 (PMC6828822; doi:10.3389/fendo.2019.00744)
Supplement: Supplementary Table 2 — Quality analysis and mapping of RNA-Seq clean reads to the rabbit reference genome. [file Table_2.DOCX]

**Suppl. Table 2. Quality analysis and mapping of RNA-Seq clean reads to the rabbit reference genome**

| **Sample** | **Number of Clean reads** | **Q20 ratio (%)** | **Q30 ratio (%)** | **GC content (%)** | **Total mapped genome (%)** | **Unique mapped genome (%)** | **Total mapped gene (%)** | **Unique mapped gene (%)** | **Total gene number** |
| --- | --- | --- | --- | --- | --- | --- | --- | --- | --- |
| C_M1 | 46858922 | 96.97 | 92.27 | 51.89 | 78.30 | 62.11 | 58.86 | 34.34 | 18476 |
| C_M2 | 48088574 | 97.09 | 92.47 | 53.02 | 77.96 | 62.44 | 59.15 | 35.14 | 18631 |
| C_M3 | 46812026 | 96.97 | 92.27 | 53.08 | 76.67 | 61.46 | 58.33 | 34.93 | 18683 |
| P_M1 | 47794818 | 97.00 | 92.31 | 53.37 | 76.70 | 60.82 | 59.83 | 35.91 | 18365 |
| P_M2 | 46797780 | 97.08 | 92.46 | 53.33 | 77.23 | 61.47 | 59.54 | 35.71 | 18440 |
| P_M3 | 48160042 | 96.84 | 91.90 | 54.69 | 75.24 | 59.27 | 60.11 | 36.78 | 18468 |
| H_M1 | 46786682 | 96.92 | 92.10 | 53.43 | 77.26 | 61.20 | 60.70 | 36.54 | 18400 |
| H_M2 | 48233750 | 96.94 | 92.16 | 53.15 | 77.56 | 61.19 | 59.99 | 35.99 | 18645 |
| H_M3 | 46790532 | 97.05 | 92.37 | 53.44 | 78.04 | 62.14 | 60.71 | 36.71 | 18499 |

C_M1, C_M2, C_M3: ovaries of rabbits just before PMSG treatment; P_M1, P_M2, P_M3: ovaries of rabbits 72 h after PMSG treatment; H_M1, H_M2, H_M3: ovaries of rabbits 48 h after hCG treatment.
