# Supplementary material for: Integrated Analysis of mRNA and miRNA Expression Profiles in the Ovary of Oryctolagus cuniculus in Response to Gonadotrophic Stimulation
Source: Front Endocrinol (Lausanne). 2019 Oct 29;10:744. doi: 10.3389/fendo.2019.00744 (PMC6828822; doi:10.3389/fendo.2019.00744)
Supplement: Supplementary Table 3 — Top 10 up/down-regulated DEGs following PMSG stimulation. [file Table_3.DOCX]

**Suppl. Table 3. Top 10 up/down-regulated DEGs following PMSG stimulation**

| **DEGs** | **Description** | **C** | **P72** | **Log_2_FC** | **FDR** | **Regulation** |
| --- | --- | --- | --- | --- | --- | --- |
| *SERPINB2* | Serpin family B member 2 | 34.0 | 568.9 | 4.1 | 4.52E-40 | Up |
| *LOC103351183* | Duodenase-1 | 7.3 | 70.4 | 3.3 | 1.06E-19 | Up |
| *SERPINE1* | Serpin family E member 1 | 460.5 | 4086.8 | 3.1 | 1.28E-29 | Up |
| *LOC100343299* | Metallothionein 2A | 81.1 | 707.2 | 3.1 | 1.13E-33 | Up |
| *PTHLH* | Parathyroid hormone-like hormone | 49.6 | 379.4 | 2.9 | 5.28E-22 | Up |
| *TIMP1* | TIMP metallopeptidase inhibitor 1 | 1474.7 | 8829.5 | 2.6 | 5.70E-77 | Up |
| *SERPINA11* | Serpin family A member 11 | 37.1 | 220.9 | 2.6 | 1.04E-12 | Up |
| *SPP1* | Secreted phosphoprotein 1 | 4973.7 | 28171.9 | 2.5 | 1.38E-53 | Up |
| *GCG* | Glucagon | 15.1 | 84.4 | 2.5 | 2.30E-10 | Up |
| *LOC108178920* | Uncharacterized | 10.8 | 60.1 | 2.5 | 7.02E-11 | Up |
| *PLP1* | Proteolipid protein 1 | 980.3 | 252.3 | -2.0 | 2.11E-15 | Down |
| *LOC100345639* | Butyrophilin-like protein 1 | 62.6 | 17.4 | -1.8 | 7.33E-06 | Down |
| *LOC103352261* | Coagulation factor XIII A chain | 1341.2 | 384.8 | -1.8 | 3.45E-18 | Down |
| *LOC100358478* | Uncharacterized | 124.8 | 36.9 | -1.8 | 1.02E-05 | Down |
| *MYBPC3* | Myosin binding protein C, cardiac | 78.5 | 25.1 | -1.6 | 1.36E-04 | Down |
| *GABRB2* | Gamma-aminobutyric acid type A receptor beta2 subunit | 278.3 | 94.0 | -1.6 | 1.63E-08 | Down |
| *CPA2* | Carboxypeptidase A2 | 48.7 | 16.7 | -1.5 | 9.01E-05 | Down |
| *WNT16* | Wnt family member 16 | 42.8 | 14.7 | -1.5 | 2.45E-04 | Down |
| *GRPR* | Gastrin releasing peptide receptor | 45.0 | 15.7 | -1.5 | 3.34E-04 | Down |
| *ABCA8* | ATP binding cassette subfamily A member 8 | 755.7 | 270.5 | -1.5 | 1.82E-07 | Down |

C, just before PMSG treatment; P72, 72 h after PMSG treatment; Log_2_FC, log_2_(P72/C); FDR, false discovery rate.
