# Supplementary material for: Integrated Analysis of mRNA and miRNA Expression Profiles in the Ovary of Oryctolagus cuniculus in Response to Gonadotrophic Stimulation
Source: Front Endocrinol (Lausanne). 2019 Oct 29;10:744. doi: 10.3389/fendo.2019.00744 (PMC6828822; doi:10.3389/fendo.2019.00744)
Supplement: Supplementary Table 4 — All up/down-regulated DEGs following hCG stimulation. [file Table_4.DOCX]

**Suppl. Table 4. All up/down-regulated DEGs following hCG stimulation**

| **DEGs** | **Description** | **P72** | **H48** | **Log_2_FC** | **FDR** | **Regulation** |
| --- | --- | --- | --- | --- | --- | --- |
| *LOC100009551* | Liver carboxylesterase | 85.1 | 400.7 | 2.2 | 2.96E-31 | Up |
| *MMP13* | Matrix metallopeptidase 13 | 28.8 | 132.3 | 2.2 | 8.86E-25 | Up |
| *PTGFR* | Prostaglandin F receptor | 188.5 | 691.9 | 1.9 | 1.51E-18 | Up |
| *SFRP4* | Secreted frizzled related protein 4 | 4148.5 | 14745.1 | 1.8 | 3.53E-35 | Up |
| *HPGD* | 15-hydroxyprostaglandin dehydrogenase | 752.5 | 2242.3 | 1.6 | 1.23E-14 | Up |
| *NMB* | Neuromedin B | 242.9 | 713.3 | 1.6 | 1.98E-13 | Up |
| *EPDR1* | Ependymin related 1 | 973.9 | 2791.1 | 1.5 | 3.07E-21 | Up |
| *PPP1R3C* | Protein phosphatase 1 regulatory subunit 3C | 311.8 | 778.5 | 1.3 | 5.59E-14 | Up |
| *ADIPOQ* | Adiponectin, C1Q and collagen domain containing | 787.9 | 1641.6 | 1.1 | 3.75E-11 | Up |
| *DOK5* | Docking protein 5 | 112.1 | 228.9 | 1.0 | 1.64E-05 | Up |
| *SLC2A12* | Solute carrier family 2 member 12 | 41.5 | 84.7 | 1.0 | 7.73E-05 | Up |
| *AKR1C5* | Aldo-keto reductase family 1, member C1 | 3488.3 | 1398.3 | -1.3 | 3.91E-10 | Down |
| *ENPP6* | Ectonucleotide pyrophosphatase/phosphodiesterase 6 | 156.8 | 73.5 | -1.1 | 1.47E-05 | Down |
| *LOC100352281* | Ribonuclease 8 | 177.0 | 85.5 | -1.1 | 3.06E-05 | Down |
| *LOC100354670* | Endoplasmic reticulum-Golgi intermediate compartment protein 2 | 279.3 | 136.0 | -1.0 | 3.12E-08 | Down |
| *LOC100357983* | BPI fold-containing family B member 4 | 188.3 | 92.6 | -1.0 | 7.35E-05 | Down |

P72, 72 h after PMSG treatment (just before hCG treatment); H48, 48 h after hCG treatment; Log_2_FC, log_2_(H48/P72); FDR, false discovery rate.
