# Supplementary material for: Integrated Analysis of mRNA and miRNA Expression Profiles in the Ovary of Oryctolagus cuniculus in Response to Gonadotrophic Stimulation
Source: Front Endocrinol (Lausanne). 2019 Oct 29;10:744. doi: 10.3389/fendo.2019.00744 (PMC6828822; doi:10.3389/fendo.2019.00744)
Supplement: Supplementary Table 5 — Categories of small RNA-Seq clean tags mapped to Rfam. [file Table_5.DOCX]

**Suppl. Table 5. Categories of small RNA-Seq clean tags mapped to Rfam**

| **Sample** | **Number of clean reads** | **Q20 ratio (%)** | **GC content (%)** | **Mapped genome (%)** | **Number of clean tags** | **Mapped genome (%)** | **Exon (%)** | **Intergenic (%)** | **Intron (%)** | **miRNA (%)** | **Precursor (%)** | **rRNA (%)** | **Repeat (%)** | **sRNA (%)** | **snRNA (%)** | **snoRNA (%)** | **tRNA (%)** | **Unmapped (%)** |
| --- | --- | --- | --- | --- | --- | --- | --- | --- | --- | --- | --- | --- | --- | --- | --- | --- | --- | --- |
| C_M1 | 13876282 | 99.73 | 44.94% | 89.54 | 1129618 | 74.80 | 5.09 | 56.32 | 8.62 | 2.99 | 0.72 | 0.48 | 5.73 | 0.13 | 0.02 | 0.37 | 0.03 | 19.50 |
| C_M2 | 13684954 | 99.71 | 45.27% | 89.54 | 1382218 | 78.49 | 4.69 | 63.36 | 6.75 | 2.27 | 0.53 | 0.38 | 6.01 | 0.09 | 0.01 | 0.35 | 0.02 | 15.54 |
| C_M3 | 13771459 | 99.70 | 45.01% | 89.90 | 1131376 | 80.50 | 5.82 | 60.78 | 8.48 | 2.97 | 0.70 | 0.39 | 6.27 | 0.10 | 0.02 | 0.32 | 0.03 | 14.13 |
| P_M1 | 13784029 | 99.64 | 44.72% | 90.59 | 1135508 | 75.26 | 4.40 | 58.40 | 7.79 | 3.23 | 0.83 | 0.52 | 6.10 | 0.15 | 0.02 | 0.48 | 0.05 | 18.03 |
| P_M2 | 13647880 | 99.63 | 44.81% | 91.00 | 1026105 | 77.44 | 4.76 | 59.78 | 7.32 | 3.38 | 0.83 | 0.69 | 5.95 | 0.17 | 0.02 | 0.44 | 0.04 | 16.62 |
| P_M3 | 13858420 | 99.72 | 44.89% | 90.58 | 1258688 | 78.28 | 5.57 | 60.25 | 8.24 | 2.77 | 0.59 | 0.38 | 5.92 | 0.10 | 0.01 | 0.26 | 0.03 | 15.88 |
| H_M1 | 13618759 | 99.69 | 44.43% | 90.83 | 822048 | 73.42 | 4.44 | 54.39 | 8.18 | 4.07 | 1.17 | 0.53 | 5.93 | 0.14 | 0.02 | 0.41 | 0.09 | 20.62 |
| H_M2 | 13609503 | 99.68 | 44.63% | 89.78 | 967214 | 74.40 | 5.05 | 54.83 | 8.50 | 3.66 | 1.02 | 0.50 | 6.16 | 0.12 | 0.01 | 0.33 | 0.07 | 19.72 |
| H_M3 | 13899066 | 99.71 | 45.11% | 91.11 | 1228010 | 79.09 | 5.18 | 61.47 | 7.54 | 2.64 | 0.58 | 0.52 | 6.39 | 0.11 | 0.02 | 0.40 | 0.03 | 15.13 |

C_M1, C_M2, C_M3: ovaries of rabbits just before PMSG treatment; P_M1, P_M2, P_M3: ovaries of rabbits 72 h after PMSG treatment; H_M1, H_M2, H_M3: ovaries of rabbits 48 h after hCG treatment
