# Supplementary material for: Integrated Analysis of mRNA and miRNA Expression Profiles in the Ovary of Oryctolagus cuniculus in Response to Gonadotrophic Stimulation
Source: Front Endocrinol (Lausanne). 2019 Oct 29;10:744. doi: 10.3389/fendo.2019.00744 (PMC6828822; doi:10.3389/fendo.2019.00744)
Supplement: Supplementary Table 6 — Top 10 up/down-regulated known miRNAs following PMSG stimulation. [file Table_6.DOCX]

**Suppl. Table 6. Top 10 up/down-regulated known miRNAs following PMSG stimulation**

| **Known miRNAs** | **C** | **P72** | **Log_2_FC** | **P Value** | **Regulation** |
| --- | --- | --- | --- | --- | --- |
| miR-451-3p | 292.8 | 18873.5 | 6.0 | 3.48E-05 | Up |
| miR-7b | 285.7 | 13373.6 | 5.5 | 7.61E-05 | Up |
| miR-144-5p_1 | 29.2 | 1098.5 | 5.2 | 2.36E-05 | Up |
| miR-129a-3p | 8.8 | 284.1 | 5.0 | 2.76E-05 | Up |
| miR-144_1 | 129.5 | 2688.1 | 4.4 | 7.17E-04 | Up |
| miR-451 | 1071.9 | 21252.9 | 4.3 | 1.27E-03 | Up |
| miR-7 | 371.1 | 6067.5 | 4.0 | 1.73E-03 | Up |
| miR-21-3p_3 | 0.4 | 5.9 | 3.9 | 3.65E-02 | Up |
| miR-30c | 783.0 | 11770.0 | 3.9 | 2.11E-03 | Up |
| miR-212-3p | 100.1 | 1245.5 | 3.6 | 2.32E-03 | Up |
| miR-34b | 36.2 | 0.6 | -5.9 | 1.37E-04 | Down |
| miR-205-5p | 101.1 | 1.8 | -5.8 | 7.10E-06 | Down |
| miR-205_1 | 54.8 | 1.9 | -4.9 | 1.61E-04 | Down |
| miR-375_2 | 2353.2 | 129.5 | -4.2 | 9.27E-04 | Down |
| miR-34b_1 | 13.4 | 1.2 | -3.5 | 1.57E-02 | Down |
| miR-450b | 2748.8 | 265.9 | -3.4 | 6.63E-03 | Down |
| miR-450a | 2379.9 | 250.8 | -3.2 | 1.14E-02 | Down |
| miR-34c | 1039.1 | 114.2 | -3.2 | 4.56E-03 | Down |
| miR-30c_1 | 15005.2 | 1662.3 | -3.2 | 1.42E-02 | Down |
| let-7i-5p | 154925.7 | 24012.2 | -2.7 | 4.93E-02 | Down |

C, just before PMSG treatment; P72, 72 h after PMSG treatment; Log_2_FC, log_2_(P72/C).
