# Supplementary material for: Integrated Analysis of mRNA and miRNA Expression Profiles in the Ovary of Oryctolagus cuniculus in Response to Gonadotrophic Stimulation
Source: Front Endocrinol (Lausanne). 2019 Oct 29;10:744. doi: 10.3389/fendo.2019.00744 (PMC6828822; doi:10.3389/fendo.2019.00744)
Supplement: Supplementary Table 7 — Top 10 up/down-regulated known miRNAs following hCG stimulation. [file Table_7.DOCX]

**Suppl. Table 7. Top 10 up/down-regulated known miRNAs following hCG stimulation**

| **Known miRNAs** | **P72** | **H48** | **Log_2_FC** | **P Value** | **Regulation** |
| --- | --- | --- | --- | --- | --- |
| miR-122-3p | 151.3 | 63029.5 | 8.7 | 6.88E-07 | Up |
| miR-122_1 | 209.4 | 49174.3 | 7.9 | 2.39E-06 | Up |
| miR-205_1 | 1.9 | 381.7 | 7.6 | 4.71E-07 | Up |
| miR-205-5p | 1.9 | 205.0 | 6.7 | 1.71E-06 | Up |
| miR-34b-3p_1 | 15.2 | 1506.2 | 6.6 | 8.86E-06 | Up |
| miR-34b | 0.6 | 49.5 | 6.3 | 1.99E-05 | Up |
| miR-34c-3p | 0.3 | 21.7 | 6.1 | 2.27E-04 | Up |
| miR-34c | 119.7 | 7958.0 | 6.1 | 5.16E-05 | Up |
| miR-206_1 | 44.1 | 1708.7 | 5.3 | 1.76E-04 | Up |
| miR-122-3p_1 | 224.6 | 7626.4 | 5.1 | 3.80E-04 | Up |
| miR-335-5p | 21.0 | 1.5 | -3.8 | 6.25E-03 | Down |
| miR-199a-5p_1 | 68444.2 | 5945.5 | -3.5 | 1.59E-02 | Down |
| miR-18a-3p_1 | 1033.4 | 96.2 | -3.4 | 8.13E-03 | Down |
| miR-542-5p | 7.2 | 0.7 | -3.4 | 3.47E-02 | Down |
| miR-92a | 27721.1 | 2910.0 | -3.3 | 1.57E-02 | Down |
| miR-107_1 | 181.3 | 19.3 | -3.2 | 6.80E-03 | Down |
| miR-302d | 8.5 | 0.9 | -3.2 | 4.81E-02 | Down |
| miR-133b-3p | 39.7 | 4.7 | -3.1 | 1.02E-02 | Down |
| let-7f_1 | 261732.3 | 30836.1 | -3.1 | 3.53E-02 | Down |
| miR-363_3 | 28463.9 | 3389.8 | -3.1 | 2.68E-02 | Down |

P72, 72 h after PMSG treatment (just before hCG treatment); H48, 48 h after hCG treatment; Log_2_FC, log_2_(H48/P72).
